# The Sanitarian

**Published:** 1875-05

**Authors:** 


*The Sanitarian* is one of our most valued exchanges, and the May number contains Proceedings of N. Y. Public Health Association, Examination in State Medicine, Milk, Seasonable Food, Ventilation for Health, Burns and Scalds, the Climates of California, Preventive Medicine, etc., etc., etc.

